# Exploring psychotic symptoms: a comparison of motor related neuronal activation during and after acute psychosis

**DOI:** 10.1186/1471-244X-12-102

**Published:** 2012-08-07

**Authors:** Luke Sheridan Rains, Gregory Fallica, Owen O’Daly, James Gilleen, Vincent Giampetro, Lucy Morley, Sukhi Shergill

**Affiliations:** 1 Institute of Psychiatry, De Crespigny Park, London SE5 8AF, UK

**Keywords:** Schizophrenia, Neuroimaging, Delusions, Hallucinations

## Abstract

**Background:**

Delusions and hallucinations are classic positive symptoms of schizophrenia. A contemporary cognitive theory called the ‘forward output model’ suggests that the misattribution of self-generated actions may underlie some of these types of symptoms, such as delusions of control – the experience of self-generated action being controlled by an external agency. In order to examine the validity of this suggestion, we performed a longitudinal functional magnetic resonance imaging (fMRI) study examining neuronal activation associated with motor movement during acute psychosis.

**Methods:**

We studied brain activation using fMRI during a motor task in 11 patients with schizophrenia and 9 healthy controls. The patient group was tested at two time points separated by 6–8 weeks.

**Results:**

At initial testing, the patient group had a mean Positive and Negative Syndrome Scale score of 56.3, and showed significantly increased activation within the left inferior parietal lobe (IPL) compared to controls. Patients reported significantly decreased positive symptoms at 6–8 week followup and IPL activation had returned to normal. Our results demonstrate that first-rank positive symptoms are associated with hyperactivation in the secondary somatosensory cortex (IPL).

**Conclusions:**

These findings lend further credence to the theory that a dysfunction in the sensory feedback system located in the IPL, and which is thought to underlie our sense of agency, may contribute to the aetiology of delusions of control.

## Background

Hallucinations and delusions are hallmark symptoms of schizophrenia. The forward output model (see [1]), proposes that certain examples of these symptoms arise from a dysfunction of the patient’s internal ‘self-monitoring’ [2,3]. In the case of delusions of control, for example, which refer to the misattribution of self-generated activity to an external source or agency, this dysfunction is thought to arise in the mechanisms underlying self-generated action. Normally, a self-generated action produces an internal efference copy. The efference copy is used in conjunction with an internally represented causal model linking actions to their sensory outcomes to give a prediction of the sensory consequences of our actions [1]. According to this model, patients with delusions of control fail to integrate the intention to act with the perception of their action, and do not consider themselves as the originator. Thus a dysfunction in our internal model has the consequence of the action being assigned to an external agency; the belief being that someone else has caused their movement.

Forward output model theories claim that when an action is identified as our own, its sensory consequence is pre-attentionally attenuated [1,4-6]. Patients fail to identify their actions in this way and consequently show less attenuation. Supporting this, patients with symptoms of psychosis have been found to demonstrate significantly less attenuation than healthy controls in behavioural studies [1,7-9]. In an earlier study, we demonstrated that patients with a diagnosis of schizophrenia applied less force than controls when asked to match a given level of force directly; while both groups were equally accurate in their application of matched given force when done indirectly, using the joystick [9]. This was in accordance with a model whereby the improved accuracy of the patient group in the direct task is secondary to lower levels of sensory attenuation contingent on dysfunctional feed forward modelling of direct self-generated actions.

Correspondingly, one would expect patients to reflect this reduction in attenuation by presenting changes in neuronal activation related to sensory feedback. In the case of a motor task, one could expect to see abnormal activation in the somatosensory cortex, since this region is believed to be responsible for processing tactile stimuli. This prediction has been met by a number of previous studies of motor tasks in patients [10-14], which have reported hyperactivation in the inferior parietal lobe (Brodmann Area 40), which includes the secondary somatosensory cortex. However, there are few such studies of longitudinal functional imaging studies investigating cortical changes associated with psychosis and motor action. Of these, most have used PET (for example [10,12]). Only a couple have used fMRI [13,14]. fMRI has a number of advantages over PET, including safety considerations, which allow for a larger number of images to be acquired in a single session, and scope for an event-related design [15], thereby substantially increasing sensitivity.

We used fMRI to examine brain activation in 11 patients during a motor task modified from [10]. The task required participants to move a joystick according to two conditions: 1) as cued and 2) based on their own choice. Using whole brain scans, by comparing these conditions we isolated the neuronal regions associated with self-initiated movement.

In order to selectively target further the mechanisms underlying psychotic symptoms, we imaged the patient group at two time points: once during an acute phase, when psychotic symptoms were maximal, and secondly following 6 to 8 weeks treatment with antipsychotic medication. This group was then compared to a control group of healthy participants with no history of psychiatric illness. Our hypothesis was that patient group would show increased IPL activation during the acute phase relative to 1) during remission and 2) the control group.

## Methods

### Participants

11 right-handed patients (9 males) diagnosed with DSM-IV schizophrenia, paranoid type, were recruited (mean age 35.4 years (SD 9.2), years in full-time education 13.5 (SD 2.1), duration of illness 12.6 years (SD 9.1), NART IQ 106.9 (SD 11.0)). All were receiving stable doses of antipsychotic medication; mean dose was chlorpromazine equivalent of 523 mg/day (SD 455 mg; eight treated with conventional and three with atypical antipsychotics). The interval between the initial and follow up measurements was 6 to 8 weeks, considered sufficient to allow for change in positive symptoms, and antipsychotic medication was kept constant. Two patients failed to attend for their second scan and so were excluded from the analysis. Symptoms were assessed using the Positive and Negative Syndrome Scale (PANSS) immediately prior to scanning on both occasions.

9 right-handed healthy control participants were scanned once. Complete data were not available for one participant due to technical problems, and so he/she was excluded from subsequent analysis. The control subjects (five males and three females) were comparable to the patients for age (mean 33.3 years; SD 7.2,t(16) = −0.50, P = 0.62) and education (mean 15.7 years; SD 3.1 t(16) = 1.78, P = 0.09).

Controls were excluded if they had a history of drug or alcohol abuse, neurological illness, head injuries, speech or hearing difficulties, or any contraindications to MRI scanning such as metal implants. All subjects provided informed consent, and ethical approval was obtained from the Institute of Psychiatry Ethics Committee.

### Task

The motor task was based closely on [10]. Participants held a joystick in their right hand and looked at a computer screen being reflected in a mirror contained in a head cage used during scanning. The task comprised three conditions: a cued condition, a spontaneous condition, and a rest condition. Each trial lasted a total of 6 seconds, comprising a 1 second presentation of the cue, followed by a 3 second response time, and a 2 second wait with a fixation cross before the next trial. Each condition was featured 20 times during the task and in randomised order, lasting for 6 minutes in total.

In the cued condition, participants were directed by a cue to move the joystick in a specific direction. A red dot, positioned in one of four possible positions (up, down, left, right), was presented on the screen. After a delay of one second the dot turned green and the participant moved the joystick in the direction corresponding to its onscreen position.

In the spontaneous condition, four red dots were presented on the screen simultaneously and once they turned green the participant moved the joystick in a direction of their choice. Participants were asked not to pre-plan their action.

The rest condition, also occurring twenty times, required the participants to stare at a fixation cross appearing on the screen without performing any movement. This condition was used as a low-level baseline during the data analysis.

### Data acquisition

Data were acquired using a 1.5 Tesla GE SignaNeuro-optimized MR System (GE, Milwaukee, WI, USA) at the Maudsley Hospital, London. A quadrature birdcage head coil was used for RF transmission and reception. Two hundred and forty T2*-weighted gradient echo planar images depicting blood-oxygen-level-dependent (BOLD) contrast were acquired from 16 non-contiguous planes parallel to the anterior commissure-posterior commissure plane [slice thickness 7.7 mm, slice gap 0.7 m, repetition time (TR) 2 s, echo time (TE) 40 ms, flip angle = 90°]. A high-resolution inversion recovery echo-planar image of the whole brain was also obtained [TE = 73 ms, inversion time (TI) = 180 ms, TR = 16 s] for subsequent registration to the standard stereotaxic space of [16].

### Image analysis

Data were analysed with the XBAM software developed at the Institute of Psychiatry, London, using a non-parametric approach (for a full description and references see http://www.brainmap.it). Experimental responses were analysed by convolving each component of the experimental design with each of two gamma variate functions (peak responses at 4 and 8 seconds respectively). The best fit between the weighted sum of these convolutions and the time series at each voxel was computed. Following this, a goodness of fit statistic was computed. This consisted of the ratio of the sum of squares of deviations from the mean image intensity (over the whole time series) due to the model to the sum of squares of deviations due to the residuals (sum of squares [SSQ] ratio). The data were then permuted using a wavelet-based method. In addition to the SSQ ratio, the size of the BOLD response to each experimental condition was computed for each individual at each voxel as a percentage of the mean resting image. Within group comparisons of experimental conditions to each contrast of interest were then computed separately for the patient and the control group. The observed and permuted SSQ ratio maps for each individual, as well as the BOLD effect size maps, were transformed into standard space using a two-stage warping procedure. Group activation maps were then computed by determining the median SSQ ratio at each voxel (over all individuals) in the observed and permuted data maps (medians are used to minimize outlier effects). The distributions of median SSQ ratios over all intracerebral voxels from the permuted data were then used to derive the null distribution of SSQ ratios. Cluster level maps for both between and within group analyses were thresholded at < 1 expected type I error 3D cluster per brain. As the cluster level threshold is set at the whole brain level, the normal, voxelwise issue of multiple comparisons does not apply. Comparisons of responses between groups or experimental conditions were performed using non-parametric analysis of variance (ANOVA). Data were fitted at each intraceberal voxel at which all subjects have non-zero data using a linear model of the type Y = a + bX + e, where Y is the vector of BOLD effect sizes for each individual, X is the contrast matrix for the particular intercondition/group contrasts required, a is the mean effect across all individuals in the various condition/group, b is the computed group/condition difference and e is the vector of residual errors. The model is fitted by minimizing the sum of absolute deviations rather than the sums of squares to reduce outlier effects. The null distribution of b is computed by permuting data between conditions (assuming the null hypothesis of no effect of experimental condition or group membership) and refitting the above model. Group difference maps were computed as described above at cluster level by appropriate thresholding of the null distribution of b, to give less than one false positive 3D cluster per image. This is a standard method for tests of this kind and it gives exact P values with minimum assumptions.

## Results

The PANSS mean total score was 56.3 (SD 16.5) at the initial scan and 48.3 (SD 6.6) at follow up with mean positive PANSS scores of 15.4 (SD 6.7) at the initial scan and 11.4 (SD 4.1) at follow up. The difference between scanning sessions was significant for the positive symptoms (t(8) = 2.33, P = 0.048), but not for the total score (t(8) = 1.68, P = 0.13).

Group maps were produced for each task for all three groups – controls, patients at T1, and patients at T2. The results showed heightened activity in the left IPL (BA 40) in patients at T1 during both cued (cluster size = 3372, p = 0.0004) and spontaneous tasks (cluster size = 3470, p = 0.0003). Activity was also detected in the left Primary Somatosensory Cortex (BA 3) for controls during both tasks.

ANOVAs were performed contrasting the cued and spontaneous tasks for each of the three groups. Maps were also contrasted between groups (patients at T1 vs. controls, patients at T2 vs. controls, and patients at T2 vs. T1) for a total of 9 comparisons.

**Figure 1:**
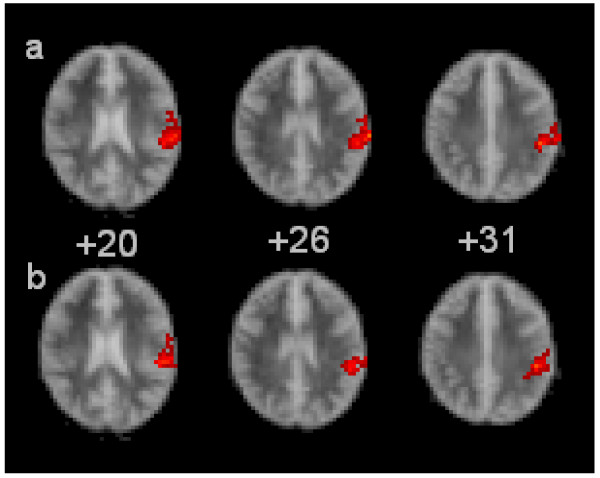
**Ascending transverse sections through the brain from left to right, relative to the intercommissural plane (mm).** The right side of the brain is shown in the left side of each image. The red clusters illustrate increased brain activation (in red) during the spontaneous task for row a) patients at T1 compared to controls and row b) patients at T1 compared to T2 In each of the images. In each case, the activation is located in BA40.

Patients at T1 demonstrated greater activation in the left IPL (BA 40) compared to both T2 (cluster size = 127, p = 0.0037) and the control group (cluster size = 119, p = 0.0039) during the spontaneous task, but not during the cued task. Greater left IPL activity in the same region was also detected during the spontaneous task compared to the cued task for both the patients at T1 (cluster size = 66, p = 0.0094) and the control group (cluster size = 416, p = 0.0002). Heightened activity was also found in the left Primary Somatosensory Cortex (BA3) in controls compared to patients at T1 in both tasks (cluster size = 90, p = 0.0031 and cluster size = 167, p 0.0029). See Figure 1.

## Discussion

We tested for differences over time in patients with schizophrenia and a group of healthy control subjects during a cued and spontaneous motor task. We found that when the patients’ PANSS positive subscale scores were maximal (i.e. when they were acutely symptomatic), they showed relative hyperactivation of the left inferior parietal lobule (BA 40) during the spontaneous condition compared to both the cued task and the other participant groups. This hyperactivity normalised 6 to 8 weeks later when the patients were in remission. These results are consistent with those from previous studies [10], and match our expectations of the IPL’s involvement in the visuomotor network.

According to [1], the forward output model states that 'any predictable signal has less impact on the nervous system (unless it has some special a priori value).' Under such circumstances, the model therefore suggests that a predictable outcome will elicit lower levels of neuronal activation in the relevant cortical region(s). We tested this hypothesis by asking participants to perform both cued and spontaneous motor tasks. The cued tasks are believed to offer greater predictability than the spontaneous tasks in terms of the expected sensory outcome they will elicit for the participants, because participants hold in their working memory the nature and direction of the arm movement beforehand. The spontaneous tasks meanwhile are intended to minimise future planning, and so correspondingly minimise predictability for the participants. Our results appear to be consistent with this expectation. The spontaneous task consistently elicited greater levels of neuronal activation in the IPL than the cued task.

The comparison of spontaneous tasks at time 1, when the patient group’s PANSS positive subscale scores were maximal, with time 2, when their symptoms had reduced, showed that the IPL was most active at time 1, while there was no significant difference in cortical activation between controls and patients at time 2. This finding contributes to the evidence supporting the efficaciousness of the forward output model in explaining certain types of delusions and hallucinations. The model predicts a lack of attenuation associated with the symptoms, and our results corroborate this by demonstrating a connection between increased activation in the IPL and increased positive symptoms during a motor task.

Our findings further demonstrate that IPL hyperactivation is systematically found in patients with positive symptoms of psychosis while performing motor activity. However, while we observed left hemisphere hyperactivation, most studies have implicated the right (e.g. [10]). Our findings indicate that psychotic symptoms may not be specifically associated with right IPL hyperaction during motor tasks, but that the hemispherical location may be more complex. It could be argued that these studies typically enrol patients specifically with delusions of control as their test participant group, and that the difference in patient group may be responsible for the difference in results. However, evidence for right hemisphere specificity comes from a range of patient groups specifically reporting psychotic symptoms, and is not restricted to patients reporting delusions of control (e.g. [17]). Meanwhile, [13] also enrolled patients diagnosed with schizophrenia and reporting high PANSS scores, but not specifically delusions of control, and reported right hemispherical hyperactivation of the IPL. Therefore, the difference in hemispherical location is unlikely to be due to the patient group. In addition we are not alone in finding hyperactivation either bilaterally or in the left IPL (e.g. [10,11,14,18,19]). Therefore, it is doubtful that the choice of patient group can account for the difference in hemispherical location, while bilateral involvement is a more reasonable conclusion.

Possible limitations for this study include the generalisability of our results, since our patient and control groups both included relatively small numbers. However, we used a non-parametric statistical approach as well as stringent thresholds for all of our analyses to minimize any Type I errors. Secondly, we cannot account for test–retest effects since the control group was scanned only once. However, this seems unlikely because differences over time were greater in the spontaneous task, which could argue against any non-specific effects of repetition. Also previous studies have not reported test-retest effects in the IPL [10]. A significant correlation between drop in patients’ PANSS scores, and particularly in the positive items, with change in IPL activation would help address this concern. However, regression analysis did not indicate a significant direct correlation between changes in symptom level and neuronal activation between scanning sessions. This may be a consequence of limited variance in the symptom change or the sample size.

The strength of this study is that medication was kept constant throughout our study. Finally, the assumption regarding the functional difference between cued and spontaneous tasks could have been tested further. Since both tasks feature self-generated movement, they both have an element of predictable sensory feedback signals. Future studies could usefully create a parameterised differential in the predictability between tasks.

## Conclusions

In summary, our findings indicate the role of hyperactivation in the somatosensory system in the neurobiology of psychotic symptoms. Specifically we found that self-generated action during a motor task specifically elicited hyperactivation in the left IPL in patients with acute psychosis. This evidence provides further support for the theory that delusions of control can be explained in terms of improperly attenuated sensorimotor feedback, as suggested by the forward output model.

## Competing interests

Dr. Shergill receives salary support from the Mental Health Biomedical Research Centre at South London and Maudsley NHS Foundation Trust and King's College London. The views expressed are those of the author(s) and not necessarily those of the NHS, the NIHR or the Department of Health.

## Authors’ contributions

SRL wrote the manuscript, FG contributed to conducting the study and analysed the data. ML., OO and GJ participating in designing and conducting the study. GV oversaw and assisted with the data analysis. SS was the PI on the project and oversaw each part. All authors read and approved the final manuscript.

## Pre-publication history

The pre-publication history for this paper can be accessed here:

http://www.biomedcentral.com/1471-244X/12/102/prepub
